# Altered central vestibular processing in Parkinson’s disease with Pisa syndrome

**DOI:** 10.1038/s41598-026-55982-z

**Published:** 2026-06-08

**Authors:** Christoph Best, Johannes Braunschädel, Felix Bethke, Anna Mueck, Iris Reuter, Heidrun H. Krämer

**Affiliations:** 1 Department of Neurology, Philipps-University, Marburg, Germany; 2 Department of Neurology, Justus-Liebig-University Giessen, Klinikstraße 33, 35392 Giessen, Germany

**Keywords:** Parkinson’s disease, Body perception, Pisa syndrome, Subjective visual verticality (SVV), Diseases, Medical research, Neurology, Neuroscience

## Abstract

In Parkinson’s disease (PD) dizziness, imbalance, postural instability and impaired visuospatial orientation occur, about 10% of PD additionally present with Pisa syndrome (PS). Aim of the current study was (a) to evaluate the perception of verticality by subjective visual vertical (SVV) and visuospatial orientation by the Benton Judgement of Line Test (JLO) in PS, (b) to analyze vestibular reactivity after vestibular stimulation and (c) to compare isolated cervical dystonia (iCD) before and after botulinum neurotoxin (BoNT) treatment. Participants consisted of 20 PD patients (8 with PS), 32 iCD patients and 51 controls. All underwent a clinical examination, evaluation of JLO and SVV. JLO and SVV were tested before and after vestibular activation by galvanic vestibular stimulation (GVS). iCD patients were treated with BoNT and performed a second trial of the study protocol. PS had pathological deviations of SVV (SVV = 2.3 ± 0.7; F = 5.22, *p* = 0.002). After GVS this deviation further increased (SVV = 3.1 ± 0.9; F = 7.36, *p* < 0.001). The course of the SVV deviation induced by GVS differed significantly (rm-ANOVA: F = 6.09, *p* = 0.003), displaying a pathological response to vestibular activation in PS only. For iCD a significant decrease in JLO was detected after GVS (JLO = 11.4 ± 0.5; F = 2.82, *p* = 0.043). Finally iCD significantly improved after BoNT treatment, without an effect on SVV. PS patients have pathological perception of verticality. Since SVV deviations further increase in PS after GVS, this points to a pathological central processing of vestibular information. Therefore, PS may be associated with involvement of central vestibular and graviceptive pathways in PD.

## Introduction

Pisa syndrome (PS) is characterized by a lateral tilt of the trunk, of more than 10°, which can at least partially be resolved by mobilization or a change in position. PS frequently manifests in Parkinson’s disease (PD), but also in other neurodegenerative conditions, and has been documented as a potential adverse medication effect^8^. The deviation of the body’s longitudinal axis cannot be explained solely by muscular weakness or skeletal deformities, but is probably based on a multifactorial pathophysiology^37^. However, a central aspect is the perception of verticality. The subjective visual vertical (SVV) and visuospatial perception play a particularly important role. Studies show that PD patients with PS more frequently exhibit deviations in SVV than those without Pisa^24^. The exact pathophysiology of PS has not yet been conclusively clarified. Various hypotheses suggest impaired multisensory integration (visual vestibular proprioceptive), asymmetric basal ganglionic dysfunction, muscular imbalance, or paraspinal dystonia^25^.

In isolated cervical dystonia (iCD) verticality and body perception also play a crucial role. iCD refers to a form of dystonia that affects only the neck muscles^1^. Patients with iCD often suffer from impaired head posture and possible impairments in spatial orientation and balance. However, studies on the perception of subjective visual verticality (SVV) showed no pathological results in iCD^12^. iCD patients exhibit a higher threshold for vestibular perception^21^. Furthermore, studies on neck proprioception suggest that neck sensorimotor processing and spatial orientation may be impaired in iCD^6^. Despite often visually intact verticality perception, iCD is associated with deficits in vestibular and proprioceptive orientation systems. These deficits appear relevant for balance, postural control, and everyday functions in affected individuals.

The aim of our study was to systematically demonstrate the relationship between PS and impaired verticality perception, to analyze the influence of vestibular galvanic activation on verticality perception. Therefore identifying and characterizing possible confounding factors such as basal ganglia dysfunction, muscular imbalance, and paraspinal dystonia.

## Methods

### Study design and participants

This prospective, monocentric, controlled study included a total of 20 PD patients, 32 patients with iCD, and 51 healthy controls (HC), matched for age and gender. PD and iCD patients were recruited from the outpatient clinic for movement disorders, and from the inpatient care, Department of Neurology, Justus Liebig University, Giessen. HC were recruited from hospital staff, through public notices, and from staff relatives.

### Study protocol

All participants underwent a detailed clinical neurological examination, including vestibular testing. Following these clinical procedures, they underwent an evaluation of the Judgement of Line Orientation test (JLO) and an examination of SVV. iCD patients additionally received an evaluation of the Toronto Western Spasmodic Torticollis Rating Scale (TWSTRS). All participants underwent a galvanic vestibular whole nerve stimulation (GVS). Hereafter, JLO and SVV were immediately tested again. The SVV was analyzed a third time with a latency of 20 min.

iCD patients were thereafter treated with botulinum neurotoxin (BoNT) injections. The target muscles were identified using clinical examination, muscle ultrasound, and electromyography. Muscle ultrasound served to identify target muscles and also to monitor injection target and success. The dose of applied BoNT was adjusted to number and size of the identified muscles and the severity of symptoms. With a latency of 6 weeks, the study protocol was re-evaluated before and after GVS.

### Diagnostic criteria

#### Parkinson’s disease (PD)

PD was diagnose in accordance with the Movement Disorder Society (MDS) clinical diagnostic criteria^29^. The diagnosis of PD was evaluated in all patients and defined as “Clinically Established PD”. The severity of disease was assessed using the Hoehn and Yahr scale. The patients were initially characterized based on the five stages^19^. Furthermore, the MDS revised version of the Unified Parkinson’s Disease Rating Scale (UPDRS) was evaluated^18^. All PD patients were examined in their clinically defined ON medication state. Dopaminergic medication was not withdrawn before testing. Additional presence of axial postural abnormalities, including camptocormia, were screened for clinically.

#### Pisa syndrome (PS)

PS was diagnosed clinically and quantified using a wall-mounted goniometer. The lateral trunk tilt criterion of at least 10° was assessed in the standing position while patients were in their clinically defined ON medication state^16,37^.

### Isolated cervical dystonia (iCD)

iCD was diagnosed according to the international guidelines: Movements follow a characteristic and repetitive pattern and can be accompanied by tremor^1,33^. Other typical clinical symptoms frequently occur: (a) pain in the neck and throat muscles, (b) muscle crampi or myogelosis, (c) sensory tricks, when symptom relief is achieved by stimulation during hand contact on the chin or cheek, (d) other accompanying symptoms, such as a concordant shoulder elevation.

To further characterize the severity of iCD, the TWSTRS was performed and the parts torticollis severity scale, disability scale and numeric pain rating scale were evaluated^9,32^.

### Clinical and vestibular examination

Each participant underwent a detailed neurological and bedside neuro-otological screening examination. This included positioning maneuvers for semicircular canal-related positional vertigo, gait and stance analysis, clinical head impulse testing, examination with Frenzel’s goggles, screening for gaze-evoked nystagmus, and a head shake test. These procedures were used to exclude clinically evident vestibular disorders prior to study inclusion. However, they were not intended to provide a comprehensive quantitative vestibular assessment, and subclinical vestibular dysfunction cannot be excluded on the basis of these bedside tests alone. In patients with isolated cervical dystonia, these maneuvers were interpreted cautiously because abnormal head posture, restricted cervical mobility, and dystonic muscle activity may affect standardized test performance. Therefore, these tests were only used as part of the clinical screening examination and not used as quantitative outcome measures, and no group-level conclusions were drawn from them.

### Judgment of line orientation test (JLO)

JLO is a neuropsychological test designed for the assessment and evaluation of visuospatial perception, with emphasis on the ability to judge angles and orientation of lines in space^4^. We administered Version E, which is a condensed and evaluated 15 item version of the original 30-item JLO^17^. Subjects are presented with a page containing two line segments of differing orientations. A semicircular configuration of eleven lines, each numbered, are presented below. Aim is to undertake a comparative analysis of the orientation of the two upper lines with the numbered lines located below, subsequently to define the corresponding numerical values. The results are given in scores, ranging from 0 to 15, while 15 represents the optimal results. Score of below 8 can be classified as pathological while scores of 8 and 9 represent a mild deficit in visuospatial orientation^39^. Results of our participants were analyzed and categorized accordingly to those normative data.

### Subjective visual vertical (SVV)

Tilts of SVV were examined for graviceptive (utricular) function, displaying potential deficits of graviceptive pathways. All subjects were seated in an upright posture. The participants looked into a translucent bucket, ensuring that their entire field of vision was covered. The bucket was positioned along the visual axis at a horizontal angle. A straight black line at the bottom of the bucket was the target pointer, and an angle scale with a centrally fixed and freely suspended plumb line was attached to the back of the bucket. The measurements were obtained from a random offset position. From the initial position, the examiner initiated a gradual rotation of the bucket, directing it towards a vertical orientation. Subjects were instructed to utilize the “stop” signal when the target pointer had reached a vertical position. The examiner initiated the test on six repetitions, three from a clockwise (CW) offset position and three from a counterclockwise (CCW) offset position, this in a pseudo randomized manner. The evaluation was performed as an overall mean and separately for each offset direction. Therefore clockwise tilting was characterized with a “plus” ( + = positive values) representing a tilt to the left from the patient`s view and counterclockwise tilting was characterized with a “minus“ (- = negative value) representing a tilt to the right from the patient`s view. Deviations of more than 2.3° (both clockwise and counterclockwise) were considered pathological^15,41^. The results were reviewed individually for every patient an direction, for group analyses averaged absolute values were entered.

### Galvanic vestibular stimulation

For galvanic vestibular stimulation (GVS), a carbon electrode was applied to the surface of the mastoid bone. To achieve the purest possible vestibular activation, without somatosensory stimulation, the skin was anesthetized with lidocaine cream. Stimulation was induced using electrical impulses with an intensity between − 4 to + 4 mA. The cathodal and anodal stimulation sides were alternated, thus stimulating both vestibular nerves. The frequency of the periodically alternating stimulation (alternating and direct current) was 1 Hz with a stimulus duration of 500 ms^22^. The stimulation results in a strong vestibular activation with the illusion of dizziness. The method is based on the neurophysiological studies of the electrical physiology of the vestibular system by Goldberg^20^. Before performing GVS, the electrical threshold for triggering subjective dizziness or vertigo was assessed in each subject. For the study protocol, stimulation was performed with a stimulus intensity + 1 mA above this threshold. Overall, GVS induced dizziness/vertigo at a mean intensity of 1.42 mA ± 0.18 mA. During the stimulation, GVS was performed at a mean stimulus intensity of 2.1 mA ± 0.23 mA. GVS was applied prior to, but not during, SVV or JLO testing. SVV and JLO were assessed at baseline and immediately after completion of GVS. SVV was additionally reassessed after a 20-minute latency period.

### Exclusion criteria

The following criteria were defined in order to exclude participants from the study: (1) Presence of a physical illness that, due to its nature and severity, could interfere with the planned examinations, influence the parameters to be examined, or endanger the subject during the examination; (2) History of chronic or acute vestibular disorders or symptoms; (3) Presence of dizziness on the day of the examination; (4) Pregnancy or breastfeeding; (5) Inability to comply with the study protocol; (6) Limitation or completely loss of legal capacity; (7) Presence of acute suicidal tendencies or danger to others; (8) Intake of vestibular suppressant medication; (9) Overall reduced general health.

### Statistical analysis

The statistical analysis was carried out using SPSS Statistics (IBM, Version 29.0). Kolmogorov–Smirnov and Levene’s tests were used to analyze for distribution and homogeneity, normality for all variables. Baseline clinical and physiological data for PD, iCD and HC were analyzed by single-factor variance analysis (ANOVA). Significant changes in variables after GVS were detected by repeated measures ANOVA, ‘disease’ (PD vs. PS vs. iCD vs. HC) as between factor. SVV, JLO, and TWSTRS were predefined main outcome measures. For each outcome, an omnibus ANOVA or repeated-measures ANOVA was first performed to test for overall group effects, time effects, or group-by-time interactions, as appropriate. Greenhouse–Geisser correction was applied in repeated-measures analyses where necessary. Post-hoc t-tests were performed only after a significant omnibus effect had been detected, in order to localize differences between groups or measurement time points. The statistical analysis of the JLO was performed in three steps: First, the absolute values were compared (rm-ANOVA) before and after GVS. In a second step, values were classified according to normative data of Woodgard into (a) normal values (JLO score of 15 − 10), (b) mild deficit (9 and 8), and (c) moderate to severe deficit (7 − 0). In a third step, the data were dichotomized into normal or pathological (normal: 15 − 10, pathological: 9 − 0).

All values are given as means ± standard error (SEM). Differences were considered significant if *p* < 0.05.

## Results

### Demographic data and clinical characteristics

With regard to the demographic data, there were no significant differences in terms of age (years ± SD: HC = 61.8 ± 1.1; PD = 65.3 ± 2.2; iCD = 61.5 ± 1.9; *p* = 0.295) and gender (number of females (%): HC = 27 (53%), PD = 8 (40%), iCD = 21 (66%); *p* = 0.192) between PD, iCD and HC, thus representing demographically comparable cohorts. In addition, an acute or past vestibular deficit could be ruled out by the clinical neuro-otological examination.

### Pisa syndrome

Eight PD patients had PS. No significant difference between PD patients with or without Pisa syndrome could be detected with respect to motor- and non-motor specific disease severity characteristics. However, a statistically significant difference was observed in the distribution of gender only (see Table 1; ANOVA: F = 9.824, dF = 19, *p* = 0.007). From eight PS patients, six had a trunk tils to the right side and two showed a trunk tilt the left. Corresponding, all right trunk tilt PS patients had a SVV deviation in counterclockwise direction and the two left trunk tilt PS patients showed SVV deviation in clockwise direction.

**Table 1:** Comparison of PD patients with and without Pisa syndrom.

Demographic and clinical data	PD (*n* = 20)	Subgroup with PS (*n* = 8)	Subgroup without PS (*n* = 12)	Statistics
Age, yrs, mean (± SD)	65.3 ± 2.2	70.3 ± 3.1	62.1 ± 2.6	n.s., *p* = 0.062
Female patients (%)	8 (40)	6 (75)	2 (17)	**F = 9**,**28; p = 0.007**
Hoehn and Yahr (± SD)	2.5 ± 0.2	2.5 ± 0.4	2.5 ± 0.2	n.s., *p* = 1.0
MDS-UPDRS I (± SD)	10.0 ± 1.2	9.0 ± 2.0	10.7 ± 1.4	n.s., *p* = 0.476
MDS-UPDRS II (± SD)	12.2 ± 1.2	14.0 ± 2.4	11.0 ± 1.0	n.s., *p* = 0.476
MDS-UPDRS III (± SD)	17.4 ± 2.8	14.0 ± 3.7	19.6 ± 4.0	n.s., *p* = 0.347
MDS-UPDRS IV (± SD)	4.7 ± 0.9	5.6 ± 1.6	4.1 ± 1.0	n.s., *p* = 0.399
MDS-UPDRS SUM I-IV (± SD)	44.3 ± 4.2	42.6 ± 6.4	45.4 ± 5.6	n.s., *p* = 0.756

The demographic data and clinical characteristics of the included PD patients comparing PD patients with and without PS are presented in Table 1.

Distribution of age did not differ between groups, the proportion of female patients, however, was larger in the PS group. No significant differences were found concerning the clinical characteristics as evaluated by the Hoehn & Yahr Scale as well as the MDS-UPDRS. Abbreviations: PD = Parkinson’s disease; PS = Pisa syndrome; MDS-UPDRS = MDS revised version of the original Unified Parkinson’s Disease Rating Scale; n.s. = no significant difference.

### Judgment of line orientation test (JLO)

Before GVS, JLO performance did not differ significantly between HC, PD patients without PS, PD patients with PS, and iCD patients (ANOVA: F = 0.97, dF = 3, *p* = 0.412; Table 2). After GVS, no significant deterioration of JLO performance was observed in either PD patients without PS or PD patients with PS. In particular, the PS group did not show a GVS-induced worsening of JLO performance, indicating that the GVS-related increase in SVV deviation in PS was not paralleled by a measurable impairment of cognitive visuospatial orientation.

**Table 2:** Judgement of Line Orientation Test.

Judgement of Line Orientation Test	HC (*n* = 51)	PD / PS – (*n* = 12)	PD / PS + (*n* = 8)	iCD (*n* = 32)	Statistics
JLO measurement BEFORE GVS					
JLO score (± SD)	12.7 ± 0.3	12.5 ± 0.6	11.6 ± 0.9	11.8 ± 0.5	n.s., *p* = 0.412
JLO category (normal/mild/pathology)	46/3/2	11/1/0	6/2/0	28/1/3	n.s., *p* = 0.299
JLO dichotomy (normal/pathology)	46/5	11/1	6/2	28/4	n.s., *p* = 0.636
JLO measurement AFTER GVS					
JLO score (± SD)	13.0 ± 0.3	12.3 ± 0.8	12.0 ± 0.8	11.4 ± 0.5	**F = 2.82**,**p = 0.043**
JLO category (normal/mild/pathology)	46/3/2	10/2/0	7/1/0	26/2/4	n.s., *p* = 0.433
JLO dichotomy (normal/pathology)	46/5	10/2	7/1	26/6	n.s., *p* = 0.693

The results of the analyses of visuospatial perception as measured by the JLO comparing PD patients with PS, PD patients without PS, iCD patients and HC are presented in Table 2.

Before application of vestibular activation by GVS, JLO results did not differ between the groups. Categorizing and dichotomizing the data also did not reveal any significant effect. After the vestibular stimulation by GVS, the iCD patients showed significantly reduced JLO scores compared to HC. Abbreviations: HC = healthy controls; PD = Parkinson’s disease; PS = Pisa syndrome; iCD = isolated cervical dystonia; JLO = Judgement of Line Orientation Test; SD = standard deviation; GVS = galvanic vestibular whole nerve stimulation; n.s. = no significant difference.

Following the GVS, a significant group effect was observed (ANOVA: F = 2.82, dF = 3, *p* = 0.043, η²=0.079; Table 2), and JLO was lower in the iCD group compared to the HC (iCD = 11.4 ± 0.5 vs. HC = 13.0 ± 0.5; *p* = 0.030). However, by categorizing the data, no significant differences were detected. Within-group pre/post comparisons did not reveal a significant GVS-induced deterioration of JLO performance in any group. However, after GVS, a significant between-group effect emerged, with lower JLO scores in iCD patients compared with HC. Thus, GVS did not induce a significant within-group decline in spatial orientation scores, but post-stimulation JLO performance differed between iCD and HC.

In conclusion, visuospatial orientation and processing prior to GVS demonstrated no significant differences across all participating groups. GVS did not induce any alterations in spatial orientation scores (SVV) within the groups. However, post-GVS, a notable discrepancy was observed between iCD and HC groups.

### Subjective visual vertical (SVV)

**Fig. 1:**
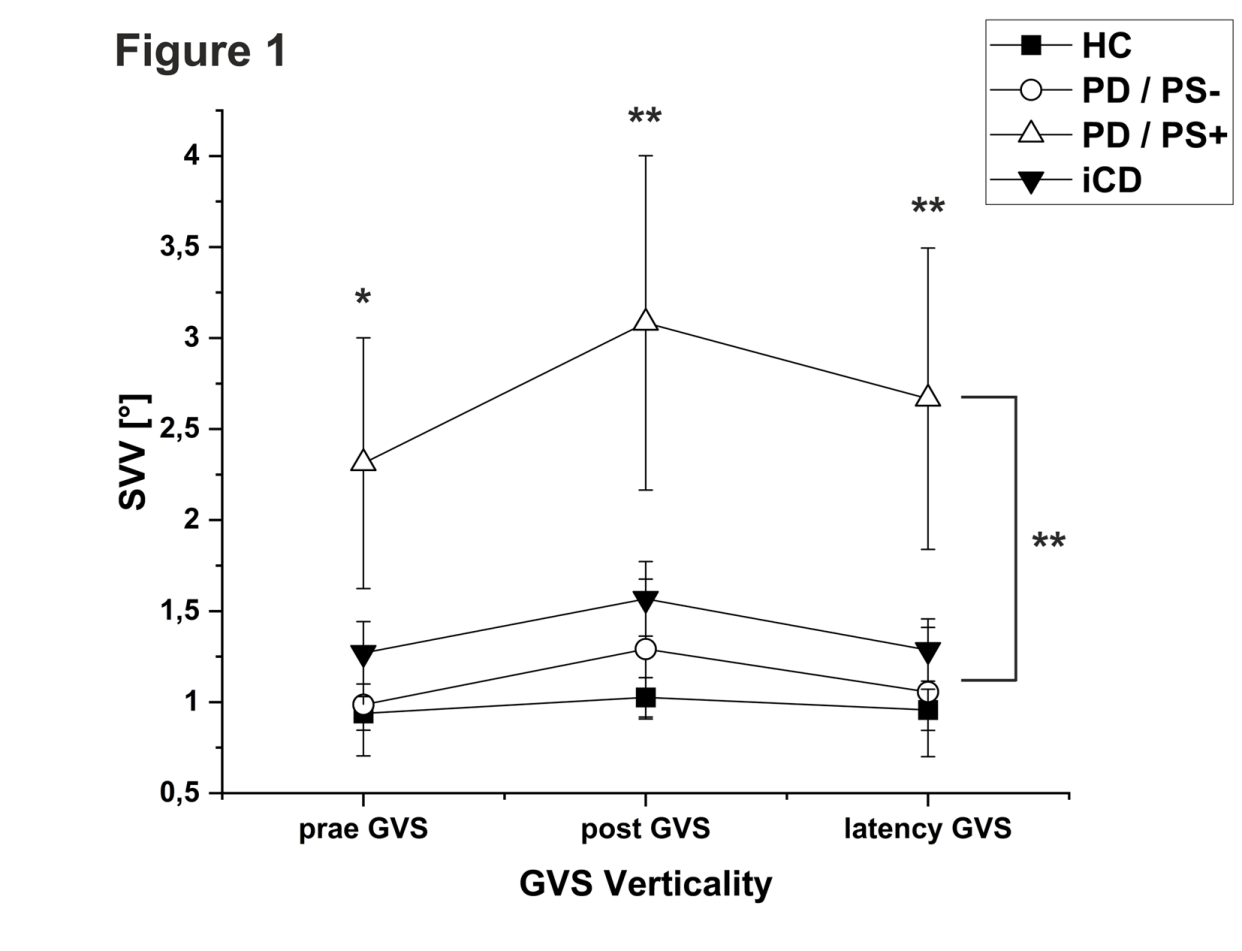
Subjective visual vertical (SVV) before and after GVS. Group mean deviations of subjective visual vertical (SVV) are shown for healthy controls (HC), Parkinson’s disease patients without Pisa syndrome (PD/PS−), Parkinson’s disease patients with Pisa syndrome (PD/PS+), and patients with isolated cervical dystonia (iCD). Measurements were performed before galvanic vestibular stimulation (pre-GVS), immediately after GVS (post-GVS), and after a 20-minute latency period (latency GVS). SVV deviations are given in degrees. Error bars indicate SEM. Abbreviations: SVV = subjective visual vertical; GVS = galvanic vestibular stimulation; HC = healthy controls; PD = Parkinson’s disease; PS = Pisa syndrome; iCD = isolated cervical dystonia. Repeated-measures ANOVA: *p < 0.01; **p < 0.001.

PD patients with PS demonstrated pathological SVV values compared to all other groups. Prior to GVS, baseline SVV values were observed to be not only significantly elevated compared to the other groups (prae GVS SVV: HC = 0.9 ± 0.1, PD = 1.0 ± 0.3, iCD = 1.3 ± 0.2, **PS = 2.3 ± 0.7; F = 5.22**,** p = 0.002;** η²=0.137), but also within the pathological range in absolute terms (SVV deviations of above 2.3°). Consequently, a pathological roll plane function was already demonstrated at baseline in PS. Following the GVS, PS patients showed further increase of pathological SVV deviation. A significant difference was observed compared to all other groups (post GVS SVV: HC = 1.0 ± 0.1, PD = 1.3 ± 0.4, iCD = 1.6 ± 0.2, **PS = 3.1 ± 0.9; F = 7.36**,** p < 0.001**, η²=0.182). Repeated measures analysis demonstrated a significant increase in pathological SVV tilt compared to baseline measurement for PS (rm-ANOVA: F = 6.09, p = 0.003). GVS induced increase of SVV deviation, with a significant difference to all other groups (rm-ANOVA: F = 7.69, p < 0.001), implying that increase of SVV deviation after vestibular activation only took place in PS. Following a 20-minute latency rest period after GVS, the pathological SVV deviations returned to the initial (pathological) level (latency GVS SVV: HC = 1.0 ± 0.1, PD = 1.1 ± 0.4, iCD = 1.3 ± 0.2, **PS = 2.7 ± 0.8; F = 5.67**,** p < 0.001**, η²=0.151). Results of SVV measurements are displayed in Fig. 1.

### Effects of botulinum neurotoxin (BoNT) injections

#### Judgment of Line Orientation test (JLO) in iCD before and after BoNT

The neuropsychological evaluation of visuospatial perception as analyzed by the JLO before vestibular activation by GVS showed no difference before and after BoNT treatment (JLO: prae BoNT = 11.8 ± 0.5 vs. post BoNT = 11.7 ± 0.6; *p* = 0.868). Similarly, the JLO after vestibular activation by GVS showed no difference before and after BoNT treatment (JLO: prae BoNT = 11.4 ± 0.5 vs. post BoNT = 12.0 ± 0.6; *p* = 0.427). Thus, the cortical and cognitive component of visuospatial perception was normal and unaltered by vestibular activation or BoNT treatment.

#### Subjective visual vertical (SVV) in iCD before and after BoNT

**Fig. 2:**
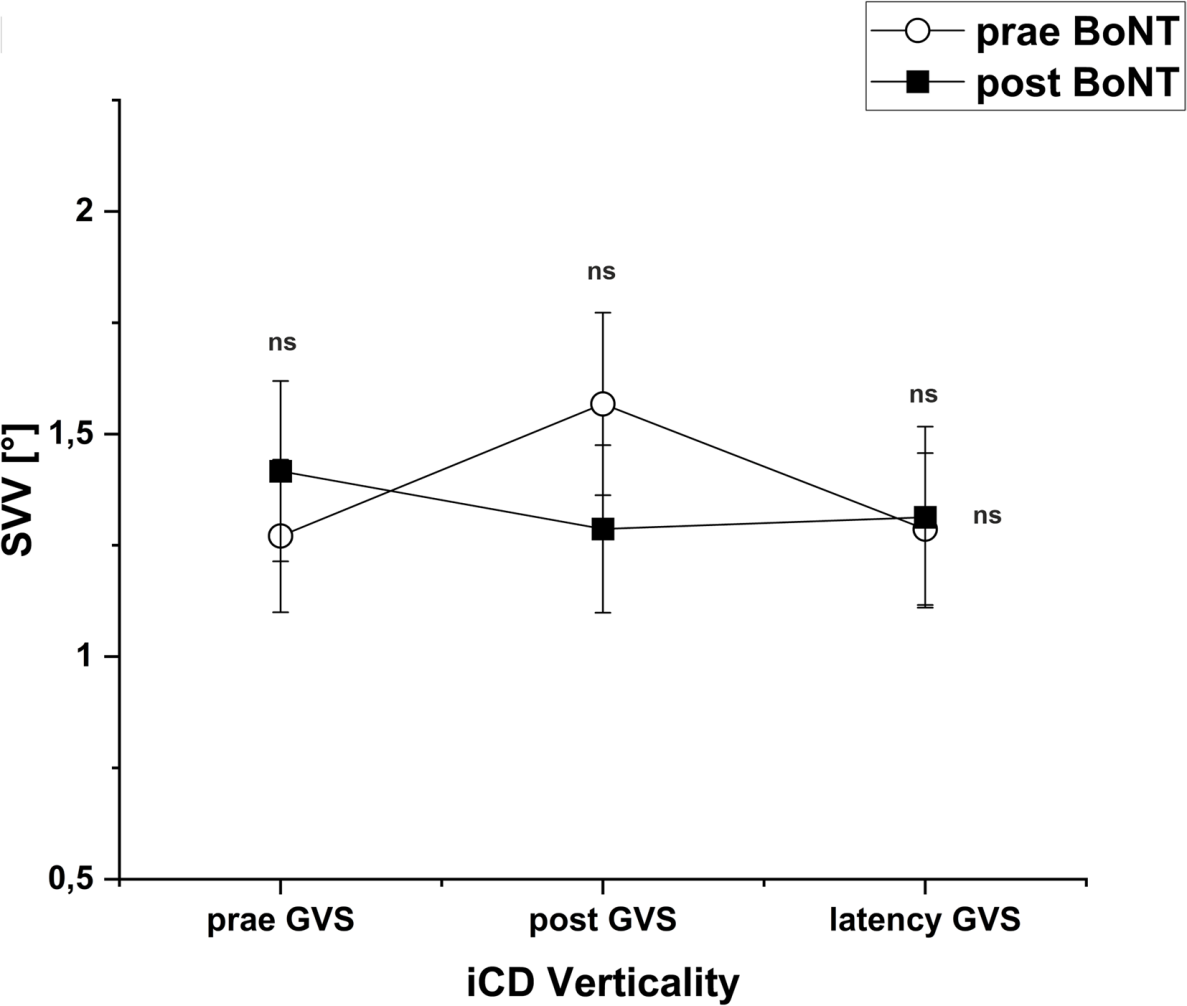
Subjective visual vertical (SVV) in iCD patients before and after BoNT treatment. Group mean deviations of subjective visual vertical (SVV) are shown for patients with isolated cervical dystonia (iCD) before and after botulinum neurotoxin treatment (pre-BoNT and post-BoNT). Measurements were performed before galvanic vestibular stimulation (pre-GVS), immediately after GVS (post-GVS), and after a 20-minute latency period (latency GVS). SVV deviations are given in degrees. Error bars indicate SEM. Abbreviations: SVV = subjective visual vertical; iCD = isolated cervical dystonia; BoNT = botulinum neurotoxin; GVS = galvanic vestibular stimulation.

Patients with iCD showed normal values for deviations of SVV throughout the whole study. Neither the GVS nor the treatment by BoNT injections had any influence on the perception of verticality or the processing of gravitational information (Fig. 2).

#### TWSTRS evaluation in iCD before and after BoNT

**Fig. 3:**
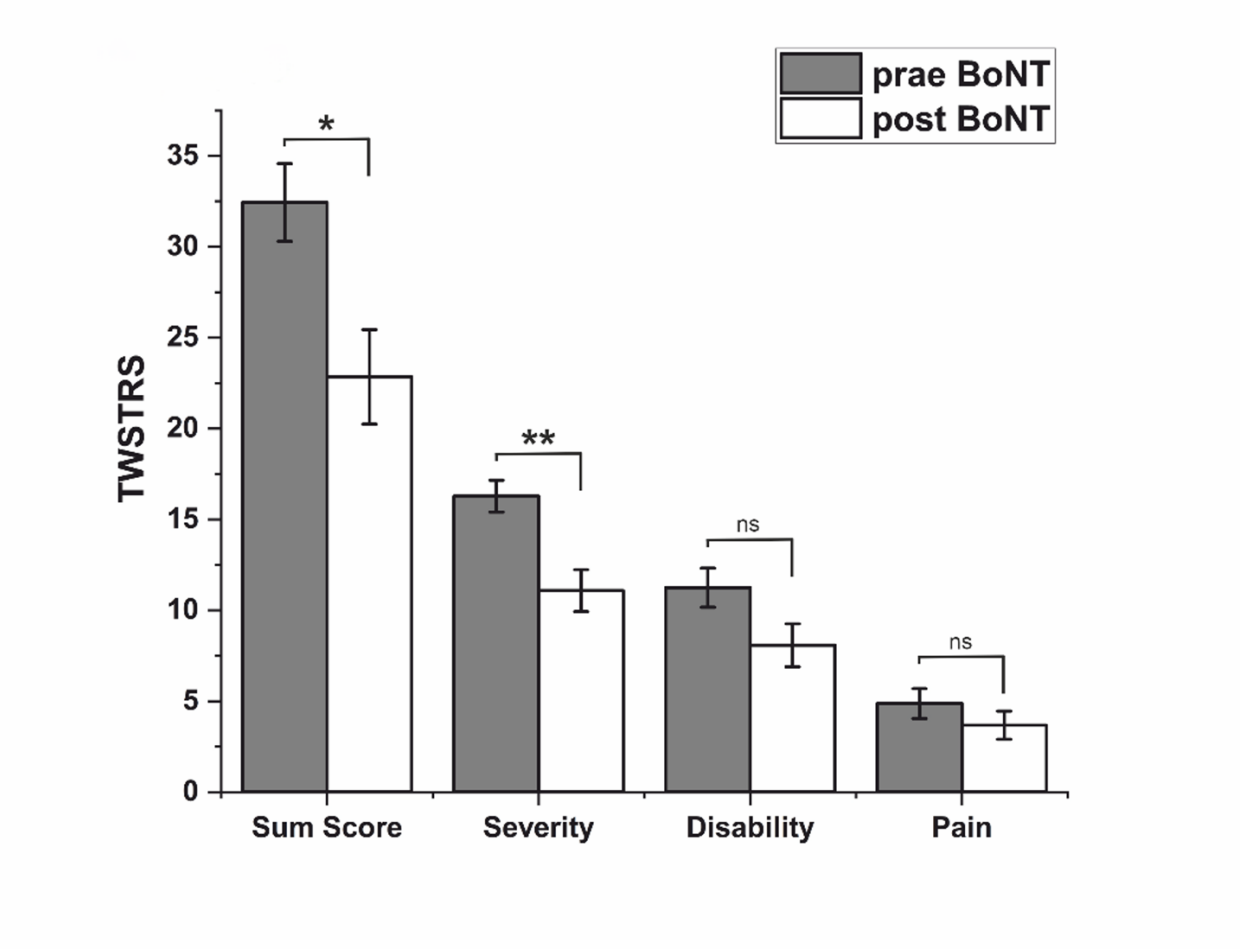
TWSTRS in iCD patients before and after BoNT treatment. Group mean values of the Toronto Western Spasmodic Torticollis Rating Scale (TWSTRS) are shown for patients with isolated cervical dystonia (iCD) before and after botulinum neurotoxin treatment (pre-BoNT and post-BoNT). Panels show (a) TWSTRS total score, (b) severity subscale, (c) disability subscale (F = 3.91, *p* = 0.053), and (d) pain subscale (F = 1.06, *p* = 0.307). Error bars indicate SEM. Abbreviations: TWSTRS = Toronto Western Spasmodic Torticollis Rating Scale; iCD = isolated cervical dystonia; BoNT = botulinum neurotoxin; ns = not significant. ANOVA: *p < 0.01; **p < 0.001.

Prior to the administration of BoNT treatment, the TWSTRS sum score for the iCD patients was 32.4 ± 2.1, and significantly decreased after treatment, reaching 22.8+- 2.6 (F = 8.30, *p* = 0.006; Fig. 3). Furthermore, the subscale “Torticollis severity scale” exhibited a significant decrease following treatment (prae BoNT = 16.3 ± 0.9 vs. post BoNT = 11.1 ± 1.2; F = 13.44, *p* < 0.001; Fig. 3). The remaining two subscales – the disability scale and the pain scale – also demonstrated a decline in score values. Nevertheless, this effect did not attain statistical significance (see Fig. 3). In the iCD cohort, head posture was clinically heterogeneous, with variable combinations of laterocollis and torticollis, sometimes occurring in the same and sometimes in opposing directions. Therefore, no single standardized measure of head tilt was available that adequately captured the complexity of dystonic head posture. The most robust clinical measure was the TWSTRS, which improved significantly after BoNT treatment, particularly in the severity subscale, with additional improvement in pain. Despite this clinical improvement, SVV remained within the normal range and was unchanged after BoNT. Thus, while musculoskeletal or proprioceptive contributions cannot be excluded in general, our findings argue against a major influence of cervical dystonia severity on SVV in the present cohort.

## Discussion

We present the first study investigating cognitive visuospatial perception and perception of verticality in PD patients with PS before and after galvanic vestibular stimulation. Results were compared with PD patients without PS, iCD patients, and healthy controls. iCD patients were examined before and after treatment with BoNT injections. PS at baseline revealed pathological deviations of visual verticality, but not of cognitive visuospatial perception. Vestibular activation further enhanced that pathological perception of verticality.

### Verticality and spatial orientation in Pisa syndrome

Research into verticality and visuospatial perception in Pisa syndrome is limited. Scocco et al. analysed SVV deviations in PD, with and without PS. They concluded that there was perceptual dysfunction or alterations of verticality^34^. Huh et al. analysed 54 patients with PD and PS. They observed a correlation between PS and SVV deviation, asymmetry of motor symptoms and advanced stage of PD. They suggested a multifactorial mechanism involving verticality perception, asymmetric basal ganglia function, vestibular hypofunction and motor disability. Sasaki et al. studied 16 patients with PD and PS. They calculated tilt thresholds in a virtual reality environment and assessed Mini-Mental State Examination (MMSE) and SVV. Tilting thresholds were significantly higher in PS and SVV performance significantly worse. They proposed a visuospatial disability in PS. Our results partly differ from those of Sasaki et al., who interpreted their results as evidence for visuospatial disability and/or attentional impairment. This discrepancy may be explained by methodological differences. The virtual reality paradigm used by Sasaki and coworkers requires complex integration of visual motion, spatial orientation, attention, and postural reference frames. In contrast, the JLO mainly assesses cognitive visuospatial line orientation, whereas SVV more directly reflects graviceptive verticality perception. The absence of significant JLO impairment in our PS group therefore does not exclude higher-order visuospatial dysfunction in PS, but suggests that the GVS-sensitive abnormality observed in our study is more closely related to vestibular verticality processing than to cognitive visuospatial line orientation. Taken together, these findings support the view that PS is multifactorial and may involve both visuospatial cognitive and vestibular-graviceptive mechanisms. Artusi et al. compared PD with and without PS, as well as PD with and without camptocormia. They examined JLO and SVV and found deficits in the visuospatial cognitive domain in association with PS. Further, Artusi et al. performed a neuropsychological examination in PD with and without PS. They found significant differences between PS + and PS- patients, with worse performance on JLO and neuropsychological parameters, postulating a link between abnormal posture and declined cognitive functions. Montse et al. studied 76 PD patients and demonstrated deficits of visuospatial function in PD.

In concordance with these studies, we detected pathological SVV deviation in PS. In contrast, however, we did not detect pathological SVV deviation in PD without PS. Furthermore, JLO was unremarkable for PD both with and without PS, which contradicts previous studies. Based on normal JLO, a cognitive component as a partial cause of impaired verticality in PS seems unlikely. Moreover, a further increase of pathological SVV deviation after GVS was observed in PS only. Apparently, PS patients suffer from a disruption of the graviceptive pathways, which cannot correctly process additional vestibular activation.

### Role of graviceptive pathways in PD

The graviceptive pathway travels from the vestibular nuclei through the medial longitudinal fasciculus to the midbrain^40^, projects to the posterolateral thalamus^3^, then to the parieto-insular vestibular cortex and other associated cortical areas^10,11^. Lesions along this graviceptive pathway induce deviations of SVV, as sensitive sign of acute vestibular imbalance in the roll plane^14^. In PD, the graviceptive pathway becomes disturbed^28^, leading to compromised perception of verticality, resulting in postural instability and an increased risk of falls. This may be due to impaired function of the otoliths, the ascending pathways, basal ganglia or thalamo-cortical networks, all of which are crucial for sensorimotor integration.

A number of studies using VEMP examinations have shown that in PD, a dysfunction of the vestibular afferents may exist^13,23,36^. Di Lazzaro and coworkers found bilateral vestibular abnormalities with bilaterally impaired cVEMPs. This cannot by itself explain the lateralized trunk tilt observed in PS. Such findings rather support a general involvement of vestibulospinal or otolith-related pathways in PD. The emergence of a lateralized Pisa phenotype probably requires additional asymmetric dysfunction, for example within basal ganglia-thalamo-cortical networks, multisensory integration, or central graviceptive processing. Our finding of pathological SVV deviation and its further increase after GVS in PS supports the concept that abnormal central processing of vestibular input may contribute to this lateralized postural disorder. Another possible explanation could lie in a deficit of cholinergic pathways to the vestibular nucleus^5^. Also, neurodegenerative changes within the vestibular nuclei have been detected, diplaying another explanation for dysfunction of graviception and postural instability^35,38^. Furthermore, decreased vestibular function in PD, evaluated by VEMP, could be modulated by levodopa administration. Pointing towards a functional aspect characterizing a levodopa dependent component of reduced vestibular excitability^30^. Finally, integration of graviceptive information and visual information is a keystone in maintaining equilibrium^7^. As neuronal activity in PD patients can be reduced within the cingulate gyrus^31^, and as motor training improved balance and postural stability in PD, resulting in an increased connectivity of visual cingulate gyrus with subcortical nuclei^26^, a dysfunction of supra tentorial vestibular areas might be a component along the graviceptive pathway, contributing to disturbances of verticality and equilibrium in PD.

In conclusion, it is evident that a potential pathological component along the ascending graviceptive pathway manifests to varying extents in PD. Consequently, it is worthwhile to discuss, whether the presence of PS in PD possibly might point towards a disease progression, encompassing functional or neuropathological impairment of vestibular function in the roll plane by affecting central graviceptive pathways.

### Role of musculoskeletal aspects

Another explanation might be musculoskeletal effects. These secondary effects may be caused by (a) the pure anatomical longitudinal body axis deviation or (b) unilaterally altered spinal muscular feedback loops.

A previous neuropsychological study demonstrated that only PD with PS exhibited pathological SVV and abnormal visuospatial performance^2^. De Pauw and colleagues analyzed SVV in iCD. No pathological result for iCD could be detected^12^. Therefore, ruling out secondary musculoskeletal effects. In accordance, we could not detect pathological SVV in iCD or PD without PS. The normal SVV findings in iCD and PD without PS argue against a major nonspecific effect of cervical dystonia or PD diagnosis alone on SVV in our cohort. However, these data do not rule out musculoskeletal or proprioceptive contributions to PS. In particular, cervical head posture in iCD and lateral trunk deviation in PS represent different biomechanical conditions and may affect graviceptive cues in different ways. Furthermore, iCD patients were treated with BoNT: TWSTRS scores improved significantly, no change in JLO or SVV was observed. Therefore, secondary effects of altered unilaterally spinal muscular feedback loops seem rather unlikely. In addition to this aspects of musculoskeletal effects, Omura et al. recently proposed that abnormal posture in PD may be related to increased muscle tone and may serve as a strategy to minimize postural sway^27^. This concept is highly relevant for the understanding of postural abnormalities in PD, particularly in the antero-posterior pitch plane. However, PS represents a lateral trunk deviation in the roll plane. Therefore, increased muscle tone and sway minimization may contribute to the maintenance or progression of abnormal posture, but do not fully explain the pathological perception of verticality observed in our PS cohort. Our data suggest that, in addition to musculoskeletal and tone-related mechanisms, impaired graviceptive processing may be specifically relevant in PS.

### Limitations

Our study however displays some limitations: First, the number of PD patients with PS was low (*n* = 8). Furthermore, vestibular evaluation complied of clinical examination and SVV evaluation. One limitation is that vestibular assessment was based on bedside neuro-otological screening and SVV measurements. Although this approach allowed us to exclude clinically evident vestibular disorders, it does not replace quantitative vestibular testing. Future studies should include vHIT, caloric irrigation, cervical and ocular VEMPs, and assessment of ocular torsion to better differentiate peripheral otolith dysfunction, vestibular nerve involvement, and central graviceptive pathway dysfunction. Another limitation concerns the clinical vestibular bedside examination in patients with isolated cervical dystonia. Head impulse testing and head shake testing may be difficult to standardize in this population, and no specific normative data are available. Accordingly, these tests were used only as screening procedures to exclude clinically evident vestibular disorders and were not included as quantitative outcome parameters. Moreover, GVS in our study protocol was applied bilaterally, thus increasing the absolute amount of vestibular activation. We did not apply unilateral GVS. Further studies could analyze, whether PS could be antagonized by GVS. PD patients were examined in the clinically defined ON medication state, and no standardized ON/OFF medication comparison was performed. Since dopaminergic medication may modulate vestibular activity in PD, medication state represents a potential confounder. Future studies should investigate SVV and GVS responses under standardized ON and OFF medication conditions. Discussing the question of disease progression markers, disease duration correlated with SVV and JLO would have been an interesting co-factor for the analyses, which we did not enter into the analyses design. This should be considered in further studies. Statistical limitations: Although post-hoc comparisons were performed only following significant omnibus ANOVA effects, multiple comparisons across the predefined outcomes and their subscales may still increase the risk of type I error. This is particularly relevant given the exploratory nature of the study and the small PS subgroup. Therefore, the findings should be interpreted cautiously and confirmed in larger independent cohorts.

## Conclusion

PD patients with PS show pathological vestibular function in the roll plane. Performing a vestibular activation by GVS, pathological SVV deviation significantly increased, identifying a pathological processing of vestibular graviceptive information. iCD patients responded significantly to BoNT treatment, by normalization of head tilt and reduction of severity, disability and pain. No pathological deviation of SVV was present in iCD patients. These findings suggest that PD patients with PS may have altered central vestibular or graviceptive processing. However, because our study was cross-sectional and included a small PS subgroup, we cannot determine whether these abnormalities reflect disease progression or whether they are directly related to falls or longitudinal postural decline.

## Data Availability

The datasets generated and/or analyzed during the current study are available from the corresponding author on reasonable request.

## Abbreviations

ANOVA: Analyses of variance
BoNT: Botulinum neurotoxin
CW: Clockwise
CCW: Counterclockwise
EMG: Electromyography
GVS: Galvanic vestibular whole nerve stimulation
HC: Healthy controls
HIT: Head impulse test
iCD: Isolated cervical dystonia
JLO: Judgement of Line Test
MDS: Movement Disorder Society
MMSE: Mini Mental State Examination
PD: Parkinson’s disease
PS: Pisa syndrome
rm-ANOVA: Repeated measures analyses of variance
SEM: Standard error of mean
SD: Standard deviation
SVV: Subjective visual vertical
TWSTRS: Toronto Western Spasmodic Torticollis Rating Scale
UPDRS: Unified Parkinson’s Disease Rating Scale
VEMP: Vestibular evoked myogenic potentials
